# Private hospital, university-led health sciences teaching and training: A proposal for the University of Limpopo’s Faculty of Health Sciences specialist lecturer shortage

**DOI:** 10.4102/jcmsa.v4i1.332

**Published:** 2026-05-30

**Authors:** Marule P. Kgagudi

**Affiliations:** 1 Department of Orthopaedic Surgery, Faculty of Health Sciences, University of the Witwatersrand, Johannesburg, South Africa

**Keywords:** medical education, graduate studies, public-private partnerships, higher education, rural health

## Abstract

Medical doctor training has largely retained the historical context of biased resource allocation. However, lately, new medical schools have been added to the lot. The University of Limpopo Medical School was established in 2016. Although successful with undergraduate training, to date, the latter struggles with both recruitment and retention of specialist staff numbers for accreditation purposes. The private sector (hospitals and staff) potentially has the resources and human capital to aid in addressing the skills shortage that exists in the province and the university. This review aims to assess the available literature and evidence that supports the role that private doctors and hospitals can play in undergraduate and postgraduate medical teaching and training. A narrative review of the literature was conducted to peruse evidence on the status of private–public partnerships in medical teaching and training. There is growing evidence for private sector teaching both locally and internationally. The government, regulators and end-users seem aligned on private sector involvement in medical training. The medical teaching and training landscape is changing with demands for innovative solutions. Infrastructure, legislation and end-users may be poised for the implementation of the private hospital, university-led health sciences teaching and training (PHUHSTT) programmes. The evidence, therefore, exists as a foundation for further engagement and possible implementation of the latter. Our proposal is not a silver bullet, but a recommendation for tackling the current state of affairs in relation to challenges in medical teaching and training at the University of Limpopo (UL).

## Introduction

The University of Limpopo’s Medical School trains medical specialists; however, it is faced with a shortage of specialists within the public sector to adequately cover all areas of teaching and learning. Private sector hospitals and practitioners may meet the required competencies (i.e. certification, clinical experience and resources) for teaching and training of future doctors and specialists alike. To our knowledge, there is no study reviewing literature on the status and willingness of stakeholders on private hospital, university-led health sciences teaching and training (PHUHSTT) in our context.

## Medical training landscape in South Africa

Current medical teaching and training in South Africa (SA) is executed by 10 established medical schools spread across six provinces, with the North West, Mpumalanga, and Northern Cape being the only three provinces without a medical school.^1^ However, the Archbishop Desmond Tutu Medical School is expected to open by the year 2028 in the North West province, making it number 11 in the country.^2^ Traditionally, medical teaching and training are typically done by public universities, hospitals and clinics.^1,3^ Private sector teaching is usually limited to minimal exposure of a few hours or months as a rotation with a private practitioner at a private hospital.^3^ However, with the increased demand in general practitioner and specialist doctor training, there is increased competition with diminishing exposure to adequate teaching.^1^ A potentially untapped area for further teaching and learning exists in collaboration with the private sector.^3^

## Proposed concept evidence

The PHUHSTT concept is gaining traction, with reports of a 250% growth in the private training of medical students in the last decade in Australia alone.^3,4^ The perceived benefits of decentralised teaching and the experiential exposure benefits within the private sector seem to be the driving force behind this approach.^5,6^ De Villers et al., in their study in SA, conducted a nationwide medical university workshop assessing factors that could contribute to successful decentralisation of medical teaching. Participants from all nine universities at the time agreed on the need for decentralisation and also noted that the chances of success were high if four recommendations were followed, namely: (1) a change in medical teaching to address malalignment in current curriculum provision; (2) involvement of medical students in that planned curriculum change; (3) the undertaking of ongoing research on the effectiveness of decentralisation; and (4) the standardisation of medical curriculum across all universities.^6^

A successful South African private medical hospital teaching collaboration exists as the WITS-Donald Gordon Medical Centre (WDGMC).^3,7^ Since its inception in 2002, the WDGMC has pioneered healthcare provision, teaching and medical research in the country.^7^ Currently, the University of the Witwatersrand registrars and undergraduate students rotate at the WDGMC for variable durations depending on their level and rotational demands. The Mediclinic Group, a private hospital group, has been involved as a partner in the establishment of both the WDGMC and a similar pilot study in collaboration with Stellenbosch University, to reproduce a similar model for the Western Cape.^3^ The latter is probably testing the potential for nationwide rollout.

## University of Limpopo Faculty of Health Sciences

The UL had its first-ever cohort of medical students enrolled in the year 2016, with the successful graduation of 47 doctors as the class of 2021.^1,8,9^ The university’s current curriculum includes general, specialist and subspecialist teaching and training.^9^ Two UL-linked teaching hospitals, namely the Polokwane Provincial and Mankweng Hospitals, lack the capacity to fully deliver on their teaching and training mandates, especially for specialists in training.^10^ Gaps in the scope of practice are currently addressed via the outreach teaching programme, which sends registrars and subspeciality candidates outside the province for augmented training. The latter is especially true in orthopaedics, psychiatry and anaesthesia departments, which have been dependent on Gauteng-based universities for assistance since the demerger with the Medical University of Southern Africa (MEDUNSA). Private hospitals and private practitioners (generalists to sub-specialists) within the provinces are equipped and educated enough to play a role in the successful implementation of PHUHSTT within the province.^3,7,10^

## The regulatory framework silver lining

The Health Professions Council of South Africa (HPCSA) deals with the accreditation of medical teaching and training as per the *Health Professions Act 56 of 1974*.^1,3,7,11^ Since the HPCSA regulates both hospitals and practitioners, whether private or public, additional regulatory requirements would include HPCSA compliance and good standing for all stakeholders with their respective associations, committees and governing policies. Private sector practitioners stand to gain continuous professional development points as well as honorary academic status with university affiliation. The overall outcome of the latter being improved compliance with the statutes of the law.^1,7^ The Hospital Association of South Africa (HASA), a body looking after the interests of private hospitals, has also expressed support for its stakeholders to get involved with health sciences teaching and training.^12,13^ In addition, the HASA has provisionally welcomed the National Health Insurance (NHI) Bill aimed at providing equitable access to both healthcare and health education in the country.^12,14^ South Africa’s new Minister of Education expressed how collaboration between the private and the public sectors is key for the future of higher education and training.^14^ He also signed into policy the recognition of private higher education institutions as ‘universities’, with the latter policy possibly paving the way for fully private medical universities in the near future.

## Impressions by stakeholders

Perceptions of both private and public patients are positive for medical teaching and training, although, with an overall paucity of literature on the topic.^4^ Australia has an established private hospital-based training and teaching programme, albeit with some room for improvement. Philips et al. noted that public hospital patients were more willing to be involved in medical teaching than their private counterparts, although this attitude seemed to be gradually improving with time.^4^ However, a qualitative survey of medical teaching conducted for surgical trainees at private hospitals revealed a variable exposure to procedures with limited scope during certain rotations.^15^ Locally on our continent, since its independence, Tanzania has run a dual system for medical training with both public and private entities providing the training of doctors to improve healthcare provision. However, challenges of unemployment and a lack of curriculum coordination highlighted flaws in the conceptual process of their model.^16^ On the other hand, the Aga Khan University of Tanzania, a fully private medical university dedicated to a holistic approach to medical education, experienced a low lecturer availability as well as resistance to curriculum change by students, also suggesting failures in prior stakeholder engagement.^17^

In SA, all medical training is currently executed at public universities; this is inclusive of the pioneering WDGMC, as it is linked to the University of the Witwatersrand (a public entity) for accreditation.^3,7^ However, the success of the latter paves the way for future advances in PHUHSTT with the possibility of a fully private medical university in the future. Private-sector practitioners (PSPs) and private patients have expressed willingness and acceptance of involvement in the private teaching of medical doctors.^18,19^ Medical students have also expressed their willingness to adopt a change in the curriculum, provided that they are consulted before any changes are implemented.^20^ A developmental problem approach with stakeholder engagement working through well-defined stages could strengthen collaboration and enhance adoption.

## Conclusion

The medical teaching and training landscape is changing with demands for innovative solutions. Infrastructure, legislation and end-users are poised for the implementation of PHUHSTT programmes. Some evidence exists which acts as a foundation for potential analysis and improvements of existing private sector-linked medical teaching and training. I propose a systematic developmental problem analysis approach with conceptualisation, implementation, monitoring and adaptation of PHUHSTT to assess its viability and potential effectiveness.
